# 4-Hydroxyisoleucine from Fenugreek (*Trigonella foenum-graecum*): Effects on Insulin Resistance Associated with Obesity

**DOI:** 10.3390/molecules21111596

**Published:** 2016-11-22

**Authors:** Anaguiven Avalos-Soriano, Ricardo De la Cruz-Cordero, Jorge L. Rosado, Teresa Garcia-Gasca

**Affiliations:** 1 Facultad de Ciencias Naturales, Universidad Autónoma de Querétaro, Juriquilla, Querétaro, Qro. 76230, Mexico; anaguiven@hotmail.com (A.A.-S.); jlrosado@prodigy.net.mx (J.L.R.); 2 Nucitec S.A. de C.V. Querétaro, Qro. 76215, Mexico; rdelacruz@nucitec.com

**Keywords:** 4-hydroxyisoleucine, *Trigonella foenum-graecum*, insulinotropic, fenugreek

## Abstract

Obesity and insulin resistance (IR) are interdependent multifactorial processes that cannot be understood separately. Obesity leads to systemic inflammation and increased levels of free fatty acids that provoke IR and lipotoxicity. At the same time, IR exacerbates adipose cell dysfunction, resulting in chronic inflammation and major lipotoxic effects on nonadipose tissues. 4-Hydroxyisoleucine (4-OHIle), a peculiar nonprotein amino acid isolated from fenugreek (*Trigonella foenum-graecum*) seeds, exhibits interesting effects on IR related to obesity. 4-OHIle increases glucose-induced insulin release, and the insulin response mediated by 4-OHIle depends on glucose concentration. The beneficial effects observed are related to the regulation of blood glucose, plasma triglycerides, total cholesterol, free fatty acid levels, and the improvement of liver function. The mechanism of action is related to increased Akt phosphorylation and reduced activation of Jun N-terminal kinase (JNK)1/2, extracellular signal-regulated kinase (ERK)1/2, p38 mitogen-activated protein kinase (MAPK), and nuclear factor (NF)-κB. Here, we present a review of the research regarding the insulinotropic and insulin-sensitising activity of 4-OHIle in in vitro and in vivo models.

## 1. Introduction

Obesity is an adverse health condition characterised by increased accumulation of body fat [1], which increases the risk of noncommunicable diseases and, in turn, can lead to death. The World Health Organization (WHO) declared obesity a global epidemic in 1997 and considers it to be the most preventable disease, principally because most obese patients benefit from changing their dietary habits and lifestyle [2]. Currently, the incidence of overweightness and obesity is dramatically rising in low- and middle-income countries due to the adoption of a lifestyle characterised by little physical activity and high consumption of hypercaloric foods [3]. Together with underweightness, malnutrition, and infectious diseases, overweightness and obesity are now considered major health problems that affect the developing world [3,4]. There is also strong evidence that obesity is associated with metabolic disorders and chronic diseases such as dyslipidaemias, hypertension, type II diabetes mellitus, cardiovascular diseases, and certain types of cancer [5,6,7,8]. 

Metabolic conditions, such as increased oxidative stress and inflammation, are part of obesity [9] and believed to be a state of low-grade chronic inflammation. As evidence of inflammation, obese individuals exhibit elevated concentrations of proinflammatory cytokines and chemokines such as interleukin-1β (IL-β), interleukin-6 (IL-6), tumour necrosis factor alpha (TNF-α), and monocyte chemoattractant protein-1 (MCP-1) [10,11]. Elevated plasma concentrations of cholesterol, triglycerides, and hormones in the renin-angiotensin system accompany these metabolic alterations [12]. Additionally, in obese individuals, adipose tissue shows chronic activation of proinflammatory signalling pathways, including Jun N-terminal kinase (JNK) and nuclear factor κB (NF-κB) [13,14]. The origin of these inflammatory mediators is hypertrophic adipose tissue. The adipocytes release proinflammatory cytokines and chemokines, recruiting monocytes to the adipose tissue and inducing the differentiation of monocytes to M1 macrophages, which produce their own proinflammatory mediators [15,16]. As a consequence, the systemic inflammation undergoes insulin resistance (IR), affecting all peripheral tissues, including adipose tissue. Thus, a vicious circle between hypertrophic adipocytes, systemic inflammation, and IR leads to obesity and related metabolic alterations. 

In order investigate the effects of 4-hydroxyisoleucine (4-OHIle) on obesity-associated insulin resistance, a review of scientific literature was conducted by searching databases in PubMed, Google Scholar, EMBASE, and the Thomson Reuters software EndNote X7.7.

## 2. Obesity, Insulin Resistance, and Lipotoxicity

**Figure 1:**
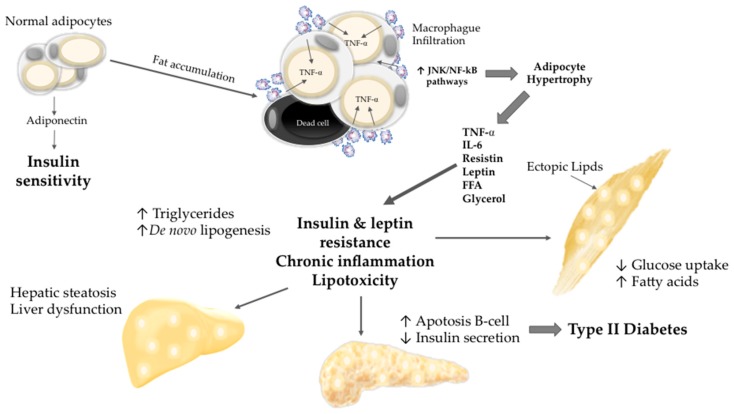
Adipocyte hypertrophy and systemic alterations. Adipocyte hypertrophy leads to chronic inflammation, insulin resistance (IR), and lipotoxicity. The increased flux of free fatty acid (FFA) to nonadipose tissues results in lipid accumulation, lipoapoptosis, and organ dysfunction.

Obesity involves adipocyte hypertrophy and hyperplasia [17,18]. Hypertrophic adipocytes release increasing amounts of proinflammatory adipocytokines such as TNF-α and IL-6, which are involved in the development of IR [19]. The decreased ability of peripheral tissues to respond to insulin leads to systemic alterations that affect both adipose and nonadipose tissues, including chronic inflammation and macrophage infiltration. The adipose and skeletal muscle cells display impaired glucose uptake, which causes hyperglycaemia. IR leads to increased lipolysis in adipocytes with a subsequent release of free fatty acids (FFAs) and glycerol. The increased flux of FFAs to other tissues such as liver, pancreas and muscle tissue results in lipid accumulation in all of these regions, exacerbating IR and other adverse effects such as lipotoxicity [20,21] (Figure 1). 

The excess of circulating FFAs in obesity causes endoplasmic reticulum stress and the activation of signalling pathways such as the JNK pathway, which promote the development of IR in obese individuals [11,22]. Due to the excess nutrients, adipocytes in obesity undergo hypertrophy, which eventually leads to adipocyte cell death. These events trigger an inflammatory response involving recruitment of macrophages to the obese adipose tissues in order to clear away dead cells [23,24]. Furthermore, high FFA levels can activate the cell surface receptor Toll-like receptor 4 (TLR4) [25,26]. The natural ligand for TLR4 is lipopolysaccharide, the Gram-negative bacterial wall component that is structurally similar to FFAs [25]. Indeed, obese individuals show upregulation of TLR4 in cells and tissues (including monocytes), adipose tissue, and skeletal muscle [27,28]. Therefore, a diet high in saturated FFAs can activate TLR4, leading to JNK activation, which in turn activates nuclear factor (NF)-κB [25,29]. Specifically, NF-κB is mainly described as playing a central role in the development of obesity and its complications such as IR, type II diabetes, and atherosclerosis [30]. The inhibitor of κB kinase (IKK)/inhibitor κB (IκB) NF-κB axis is activated by various exogenous and endogenous molecules, including FFAs, cytokines, glucose, and reactive oxygen species [31]. NF-κB regulates the expression of several proinflammatory cytokines, enzymes, adhesion molecules, and immune receptors [32].

High levels of extra-adipose FFAs lead to triglyceride accumulation in nonadipose tissues. Triglyceride levels must remain within a very narrow range in nonadipose cells; therefore, when adipose tissue systematically releases large amounts of FFAs, the increased storage of triglycerides together with IR lead to cell death, toxic effects, and impairment of the functions of nonadipose tissues. FFA deposition in nonadipose tissues is highly regulated by leptin, which participates as an antisteatotic hormone. At the beginning of the metabolic alteration, leptin signalling is normal; however, chronic obesity can provoke postreceptor leptin resistance, resulting in triglyceride accumulation and lipotoxicity [33]. As a result, an increase of de novo ceramide production in beta-pancreatic cells leads to nitric oxide-mediated lipoapoptosis, pancreatitis, and pancreatic necrosis [34,35], which results in lower blood insulin levels. Adiposopathy, the overproduction of proinflammatory adipocytokines and FFA release, together with increased recruitment of macrophages and other immune cells, provoke endothelial dysfunction and increase cardiovascular risk [36], since cardiac lipid accumulation is associated with cardiomyopathy [21]. High FFA levels impair liver metabolism of glucose, and lipotoxicity results in nonalcoholic steatohepatitis [37]. On the other hand, in muscle cells, the phosphatidylinositide 3-kinase (PI3K) signalling pathway fails, causing atypical protein kinase C (PKC) isoform activation, which contributes to IR [20,21]. FFAs can also cause toxicity to proximal tubular epithelial cells in the kidney [38].

## 3. Fenugreek (*Trigonella foenum-graecum* L.)

The optimal strategy for reversing obesity must consider the early stages of IR, and changes in lifestyle are also crucial [39]. Physical activity increases metabolic rate and energy expenditure [40]; however, this approach is short-term in duration and fat accumulation is usually observed [39]. Management of obesity usually involves a combination of lifestyle modification and some pharmacological therapy. Surgical interventions, although effective in some circumstances, are not always appropriate [41,42]. An alternative strategy seems to be the development of therapeutic agents for body weight reduction by decreasing food consumption or absorption, increasing energy expenditure, or both [43,44]. It is highly desirable that antiobesity drugs produce sustained weight loss with minimal side effects. Unfortunately, despite the short-term benefits observed, pharmacological treatment of obesity is associated with weight gain rebound after treatment completion, with undesirable side effects [42].

Throughout history, plants have been used as traditional natural medicines for curing many diseases, and they offer safe alternatives to synthetic drugs [45,46]. Naturotherapy seems to be an outstanding alternative strategy for obesity treatment and control [47,48,49]. One plant in particular, *Trigonella foenum-graecum*, or fenugreek (in Arabic, *hulabah*), has been used in Ayurvedic and Chinese medicine for numerous indications. Animal and human studies have demonstrated the hypoglycaemic and antihyperlipidemic properties of oral fenugreek seed powder, which are due to the antidiabetic compounds present in the seeds, leaves, and different type of extracts [50,51,52].

Fenugreek belongs to the family Fabaceae, and it is cultivated as a food crop in India, the Mediterranean region, North Africa, and Yemen. The seeds exhibit pungent aromatic properties [53]; fenugreek is used as a spice in curry preparations [54], to flavour food, and to stimulate appetite. It has been observed that chronic oral administration of an ethanol extract of fenugreek (10 mg/day per 300 g body weight) increases food intake in rats, possibly due to the aromatic properties of the seeds [55,56]. Fenugreek seeds are used in India, Egypt, and Yemen as a condiment and supplement in food, and its green leaves are widely consumed in India [57]. The seeds are a good source of protein, but they also contain unavailable carbohydrates, mucilages, and saponins [58,59,60,61]. Three steroidal sapogenins (diosgenin, gitogenin, and tigogenin) were reported by Anis and Aminuddin (1985) [62], and 10 different sapogenins have been identified by analytical methods including coupled GC–MS [63,64]. The biological properties of fenugreek saponins have been assessed [65] and they include hypocholesterolemic and antifungal activity as well as enhancement of food intake and feeding behaviour in rats [66,67]. Among other alkaloids, trigonelline is found in the seeds [68]. The seed contains a greater amount of minerals (Ca, P, Fe, Zn, and Mn) than other legumes [69]. The lipid content of the seed (neutral lipids, glycolipids, and phospholipids) is approximately 7.5% [70]. The aromatic constituents of fenugreek seeds include *n*-alkanes, sesquiterpenes, and oxygenated compounds such as hexanol and γ-nonalactone [56]. The seeds also contain flavonoids, carotenoids, coumarins, and other components [71]. The amino acid content is high in arginine, alanine, and glycine, but not in lysine [72]; however, the nonprotein amino acid 4-hydroxyisoleucine (4-OHIle) is abundant in the seeds [73].

Fenugreek leaves and seeds are used for medicinal purposes in many countries. Their beneficial effects include carminative, tonic, and aphrodisiac effects [74]; a stimulating effect on the digestive process [75]; hypoglycaemic effects through stimulation of insulin secretion in a glucose-dependent manner; and inhibition of α-amylase and sucrase activity [76]. Curative gastric antiulcer action by the seed [77] and hypocholesterolemic effects [78,79] have also been reported.

Evidence from animal and human studies suggests that, with regard to the biological properties of fenugreek seed powder, it is hypoglycaemic and anti-hyperlipidemic when taken orally. Fenugreek seed contains 30% soluble fibre and 20% insoluble fibre, which can slow the rate of postprandial glucose absorption, possibly as a secondary mechanism for the hypoglycaemic effect. Doses ranging from 2.5 g to 15 g daily of crushed and defatted seeds have been used in clinical studies (crushing allows for the release of the viscous gel fibre that contributes to fenugreek’s efficacy), while the seeds have been used in the range of 1–3 g mixed with food. Diarrhoea and flatulence are the most common side effects observed, and the fibre can affect absorption of oral medications. As one of the major effects of fenugreek is decreased blood glucose, careful monitoring of glucose levels is needed when it is taken concomitantly with insulin or other glucose-lowering agents. Fenugreek can also exhibit anticoagulant activity; therefore, it should be used under close medical supervision when anticoagulant agents are prescribed [80,81,82,83]. A decrease of serum triglycerides (TGs), total cholesterol, and low-density lipoprotein cholesterol (LDL-C) is observed with fenugreek seed administration. This may be due to the presence of sapogenins, which increase biliary cholesterol excretion, resulting in reduced serum cholesterol levels [84,85]. The U.S. Food and Drug Administration, with regard to food ingredients, has determined that fenugreek seed extracts are not genotoxic (based on a minimum content of 40% 4-OHIle) [86].

Fuller and Stephens (2015) summarised the evidence on the physiological effects of three bioactive compounds of fenugreek: diosgenin, 4-hydroxyisoleucine (4-OHIle), and the soluble dietary fibre fraction, with emphasis on the biological mechanisms of action underlying metabolic syndrome. The insulinotropic properties of 4-OHIle suggest its potential as an antidiabetic pharmacological compound. This could potentially overcome a common drawback of sulfonylureas, which carry the risk of inducing hypoglycaemia [87]. Gong et al. (2016) conducted a meta-analysis regarding the overall effects of fenugreek on hyperglycaemia and hyperlipidaemia in prediabetes and diabetes. In their study, fenugreek showed hypoglycaemic effects and total cholesterol-lowering efficacy; however, the effects on TGs, LDL cholesterol, and high-density lipoprotein cholesterol (HDL-C) were not confirmed, and further studies are needed [88].

## 4. Pharmacological Effects of 4-Hydroxyisoleucine (4-OHIle)

**Figure 2:**
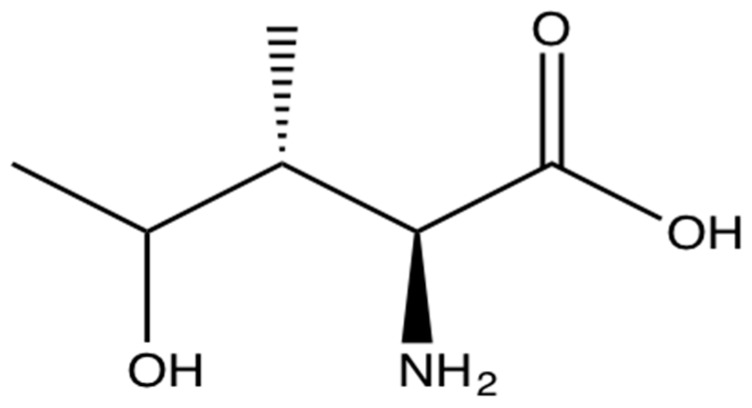
The branched-chain amino acid (2*S*,*3R*,*4S*)-4-hydroxyisoleucine.

4-OHIle is a branched-chain amino acid (Figure 2) only present in plants. It is particularly abundant in fenugreek seeds (0.015%–0.4%) [89,90,91]. It is synthesised from isoleucine and has been postulated as one of the molecules responsible for the antidiabetic effects in animals because of its ability to regulate pancreatic insulin secretion [92,93,94,95], hence it has significant potential for the treatment of IR and diabetes [96] (Table 1). 

The antidiabetic properties of 4-OHIle are related to its ability to stimulate insulin secretion, as observed in human pancreatic islet cells, in isolated perfused rat pancreas [73] and in in vivo studies [82]. An improvement in glucose and insulin tolerance, insulin secretion, and reduced hyperglycaemia were observed in diabetic rats and dogs. 4-OHIle functioned as an insulin secretagogue, but only in the presence of elevated blood glucose concentrations, in a range of 8.3–16.7 mM [73,82,83].

4-OHIle shows in vitro insulinotropic activity related to the glucose concentration of the medium in isolated pancreatic beta cells [82,83]. Furthermore, this effect has not been observed for sulphonylureas, treatment with which has produced hypoglycaemia as an adverse effect in non-insulin-dependent diabetes mellitus patients. The secretagogue potential of 4-OHIle is of special interest for various degrees of insulin resistance [97]. In streptozotocin-treated rats, an improvement of the diabetic state was associated with the stimulating effect of 4-OHIle on beta cell function, and in normal rats and dogs, 4-OHIle is able to stimulate insulin secretion and improve glucose tolerance, suggesting its potential in the treatment of IR and type II diabetes [76,89].

Reversing defective insulin secretion is highly desirable in a diabetic state, but enhancing insulin sensitivity in hepatic and peripheral tissues is also important. Insulin-sensitising studies for 4-OHIle demonstrated its efficacy in two rat models [95]. Using the hyperinsulinaemic clamp method, improvement in insulin sensitivity was observed in sucrose plus lipid-fed rats, in which peripheral glucose uptake was increased, and also in Zucker *fa/fa* rats, in which hepatic glucose output was decreased. Injection of 4-OHIle resulted in the activation of insulin receptor substrate-1 (IRS-1) and phosphatidylinositide 3-kinase (PI3K) in insulin-sensitive tissues. In in vitro assays using human and rat pancreatic cells, 4-OHIle showed increased glucose-induced insulin release, and levels of somatostatin and glucagon were not altered [92]. Cellular glucose uptake in muscle and adipose tissues is dependent on insulin stimulation, which causes the translocation of glucose transporter-4 (GLUT-4) to the plasma membrane. When insulin is deficient or its signalling pathway is altered, the translocation of GLUT-4 does not take place efficiently, resulting in decreased uptake of glucose by adipocytes and muscle cells, which in turn contributes significantly to high blood glucose levels. In fact, the restoration of GLUT-4 will produce normoglycaemia. In experimental diabetes tests it has been observed that *Trigonella* molecules are able to reverse the effects on the GLUT-4 transporter to normal levels [98].

Shukla and Rangari [99] studied the antidiabetic activity of 4-OHIle in combination with the natural bioavailability enhancers piperine and ginger oleoresin. Alloxan-induced diabetic rats were treated with fenugreek seed powder containing 28% 4-HOIle, alone or combined with the bioenhancers, producing significant blood glucose level and body weight improvement when compared to the diabetic control. Narender et al. [90] studied the effect of 4-OHIle on dyslipidaemic hamsters. 4-OHIle resulted in decreased plasma triglycerides, total cholesterol (TC), and FFAs, and a simultaneous increase by 39% of the HDL-C:TC ratio. Haeri et al. (2009) [100] determined the effect of 4-OHIle on streptozotocin-induced diabetic and fructose-fed rats. Liver function markers and glycaemia improved after an eight-week treatment at a dose of 50 mg/kg. In fructose-fed rats, blood glucose and markers of liver aminotransferases were restored to levels near those observed in control animals. In streptozotocin-diabetic rats an increase in serum HDL-C was observed. Effects of 4-OHIle were also observed in leptin receptor-deficient *db/db* mice, with improvement in levels of blood glucose, insulin, and lipids [101]. Streptozotocin-diabetic rats showed decreased blood glucose and restored blood lipid and uric acid levels after four weeks of treatment with 4-OHIle [102].

The molecular mechanism of action of 4-OHIle has been studied using cell culture models. In rat muscle cells, it has been observed that glucose uptake and GLUT-4 translocation to the plasma membrane were increased after 16 h exposure to 4-OHIle [103]. The basal phosphorylation of Akt (Ser-473) increased after treatment with 4-OHIle, but mRNA expression of total Akt, IRS-1, GLUT-4, and glycogen synthase kinase-3β (GSK-3β) was unchanged. Treatment of L6 myotubes with 4-OHIle decreased IR induced by FFAs [104]. In fact, 4-OHIle restored glucose uptake and GLUT-4 translocation to the plasma membrane after palmitate treatment via insulin induction of IRS-1 phosphorylation. 4-OHIle also inhibited both the production of reactive oxygen species induced by palmitate and the associated inflammation, and it reduced activation of the JNK1/2 pathway, including the extracellular signal-regulated kinase isoforms 1 and 2 (ERK1/2), p38 MAPK, and NF-κB. The effects of 4-OHIle on 3T3-L1 adipocytes included increased glucose uptake in insulin-resistant adipocytes in a dose-dependent manner along with a reduction of TNF-α mRNA expression and secretion, suggesting its anti-inflammatory potential [105]. 

**Figure 3:**
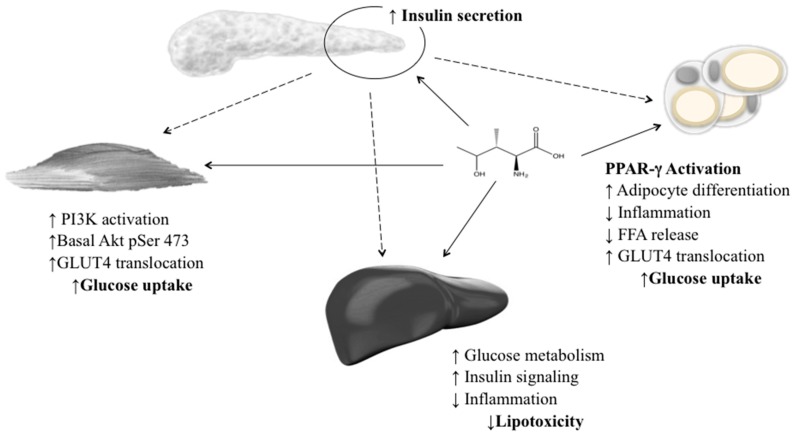
Insulinotropic effects of 4-hydroxyisoleucine in the pancreas, skeletal muscle, adipose tissue and liver. 4-OHIle increases insulin secretion and improves insulin sensitivity in nonadipose tissues.

Gao et al. (2015) established an IR HepG2 cell line and determined the molecular mechanisms for 4-OHIle in IR. Two potential mechanisms were described: a negative regulation of TNF-α production with an improvement in insulin sensitivity, and increased expression of p-IRS-1 and GLUT4 in the insulin-signalling pathway [106]. 4-OHIle has been reported as a glucose-dependent insulinotropic compound through its direct effect on pancreatic islets and its insulin-sensitising effect on muscle, adipose and liver tissue (Figure 3). These effects, in combination with the absence of acute toxicity or genotoxicity, suggest that this amino acid has a potential role as a natural product for the treatment of obesity and IR [88,107].

## 5. Conclusions 

The peculiar nonprotein branched amino acid 4-hydroxyisoleucine has been described as an efficient compound in the regulation of insulin secretion. Due to the fact that it acts as an insulin secretagogue in the presence of elevated blood glucose concentrations, it has been proposed for the potential treatment of insulin resistance, diabetes and obesity. The beneficial effects observed are related to the regulation of blood glucose, the reduction of lipotoxicity by decreasing plasma triglycerides, FFAs, and total cholesterol, and the improvement of liver function. The mechanism of action is related to a reduced activation of JNK and NF-κB activity. 4-OHIe has demonstrated unique therapeutic potential against insulin resistance and the harmful effects associated with inadequate glucose uptake, lipotoxicity, and liver dysfunction, which ultimately cause diabetes, one of the major pandemic diseases worldwide.

## Figures and Tables

**Table 1:** Effects of 4-hydroxyisoleucine in insulin resistance and its adverse metabolic effects.

	Model	Experimental results	Reference
In vitro studies	Isolated human and rat pancreas	Increase of glucose-stimulated insulin secretion	[89]
	Normal and type II diabetic rats, isolated rat islets	Increased oral glucose tolerance, increased glucose-stimulated insulin secretion	[82]
	Isolated rat islets	Increased glucose-stimulated insulin secretion	[83]
	Skeletal muscle (L6 myocytes)	Increased glucose uptake, increased Akt phosphorylation on Ser 473 (pAkt), increased PI3K, increased glucose transporter 4 (GLUT4)	[103]
	Insulin-resistant skeletal muscle (L6 myocytes)	Increased insulin receptor substrate-1 tyrosine phosphorylation (pIRS-1), increased PI3K, increased pAkt, decreased reactive oxygen species (ROS), decreased NF-kB, decreased c-Jun N-terminal kinase 1/2 (JNK), decreased p38 MAPK	[104]
	Insulin-resistant 3T3-L1 adipocytes	Decreased TNF-α, increased glucose uptake	[105]
	Insulin-resistant HepG2 cells	Reduced TNF-α, stimulated expression of IRS-1 and GLUT4, inhibited expression of p-IRS-1 (Ser307)	[106]
In vivo studies	Zucker *fa/fa* rats, high fat + sucrose fed rats	Increased glucose tolerance, increased insulin sensitivity, reduced hepatic glucose production, increased phosphatidylinositide-3 kinase (PI3K), reduced fasting insulin	[93]
	Hamsters	Decreased serum triglycerides, decreased serum total cholesterol, decreased free fatty acids, increased HDL:TC ratio	[95]
	Type II diabetic rats	Decreased blood glucose, increased serum HDL, decreased alanine aminotransferase, decreased aspartate aminotransferase	[100]
	C57BL/db/db mice	Decreased blood glucose, decreased serum triglycerides, total cholesterol and LDL, increased serum HDL	[101]
	Type I diabetic rats	Decreased blood glucose, decreased serum total cholesterol, decreased serum LDL, decreased serum triglycerides, increased serum HDL	[102]

## References

[1] World Health Organization (2009). Obesity and Overweight. http://www.who.int/dietphysicalactivity/publications/facts/obesity/en/.

[2] Caballero B. (2007). The global epidemic of obesity: An overview. Epidemiol. Rev..

[3] Hossain P., Kawar B., El Nahas M. (2007). Obesity and Diabetes in the Developing World—A Growing Challenge. N. Engl. J. Med..

[4] Haslam D.W., James W.P. (2005). Obesity. Lancet.

[5] Yang C.P., Lin C.C., Li C.I., Liu C.S., Lin W.Y., Hwang K.L., Yang S.Y., Chen H.J., Li T.C. (2015). Cardiovascular Risk Factors Increase the Risks of Diabetic Peripheral Neuropathy in Patients with Type 2 Diabetes Mellitus: The Taiwan Diabetes Study. Medicine.

[6] Reeves A.F., Rees J.M., Schiff M., Hujoel P. (2006). Total body weight and waist circumference associated with chronic periodontitis among adolescents in the United States. Arch. Pediatr. Adolesc. Med..

[7] Arner P. (2005). Insulin resistance in type 2 diabetes—Role of the adipokines. Curr. Mol. Med..

[8] Plutzky J. (2000). Emerging concepts in metabolic abnormalities associated with coronary artery disease. Curr. Opin. Cardiol..

[9] Vincent H.K., Innes K.E., Vincent K.R. (2007). Oxidative stress and potential interventions to reduce oxidative stress in overweight and obesity. Diabetes Obes. Metab..

[10] Bastard J.P., Maachi M., Lagathu C., Kim M.J., Caron M., Vidal H., Capeau J., Feve B. (2006). Recent advances in the relationship between obesity, inflammation, and insulin resistance. Eur. Cytokine Netw..

[11] Hotamisligil G.S. (2006). Inflammation and metabolic disorders. Nature.

[12] Apostolopoulou M., Savopoulos C., Michalakis K., Coppack S., Dardavessis T., Hatzitolios A. (2012). Age, weight and obesity. Maturitas.

[13] Olefsky J.M., Glass C.K. (2010). Macrophages, inflammation, and insulin resistance. Annu. Rev. Physiol..

[14] Wellen K.E., Hotamisligil G.S. (2005). Inflammation, stress, and diabetes. J. Clin. Investig..

[15] Weisberg S.P., McCann D., Desai M., Rosenbaum M., Leibel R.L., Ferrante A.W. (2003). Obesity is associated with macrophage accumulation in adipose tissue. J. Clin. Investig..

[16] Xu H., Barnes G.T., Yang Q., Tan G., Yang D., Chou C.J., Sole J., Nichols A., Ross J.S., Tartaglia L.A. (2003). Chronic inflammation in fat plays a crucial role in the development of obesity-related insulin resistance. J. Clin. Investig..

[17] Couillard C., Mauriège P., Imbeault P., Prud’homme D., Nadeau A., Tremblay A., Bouchard C., Després J.-P. (2000). Hyperleptinemia is more closely associated with adipose cell hypertrophy than with adipose tissue hyperplasia. Int. J. Obes. Relat. Metab. Disord..

[18] Konturek P.C., Konturek J.W., Cześnikiewicz-Guzik M., Brzozowski T., Sito E., Konturek S.J. (2005). Neuro-hormonal control of food intake: Basic mechanisms and clinical implications. J. Physiol. Pharmacol..

[19] Kahn S.E., Hull R.E., Utzschneider K.M. (2006). Mechanisms linking obesity to insulin resistance and type 2 diabetes. Nature.

[20] Kahn B.B., Flier J.S. (2000). Obesity and insulin resistance. J. Clin. Investig..

[21] Schaffer J.E. (2003). Lipotoxicity: When tissues overeat. Curr. Opin. Lipidol..

[22] Ozcan U., Cao Q., Yilmaz E., Lee A.H., Iwakoshi N.N., Ozdelen E., Tuncman G., Görgün C., Glimcher L.H., Hotamisligi G.S. (2004). Endoplasmic reticulum stress links obesity, insulin action, and type 2 diabetes. Science.

[23] Rutkowski J.M., Davis K.E., Scherer P.E. (2009). Mechanisms of obesity and related pathologies: The macro- and microcirculation of adipose tissue. FEBS J..

[24] Trayhurn P., Wood I.S. (2004). Adipokines: Inflammation and the pleiotropic role of white adipose tissue. Br. J. Nutr..

[25] Shi H., Kokoeva M.V., Inouye K., Tzameli I., Yin H., Flier J.S. (2006). TLR4 links innate immunity and fatty acid-induced insulin resistance. J. Clin. Investig..

[26] Suganami T., Tanimoto-Koyama K., Nishida J., Itoh M., Yuan X., Mizuarai S., Kotani H., Yamaoka S., Miyake K., Aoe S. (2007). Role of the Toll-like receptor 4/NF-kappaB pathway in saturated fatty acid-induced inflammatory changes in the interaction between adipocytes and macrophages. Arterioscler. Thromb. Vasc. Biol..

[27] Reyna S.M., Ghosh S., Tantiwong P., Meka C.S., Eagan P., Jenkinson C.P., Cersosimo E., DeFronzo R.A., Coletta D.K., Sriwijitkamol A. (2008). Elevated toll- like receptor 4 expression and signaling in muscle from insulin-resistant subjects. Diabetes.

[28] Jialal I., Huet B.A., Kaur H., Chien A., Devaraj S. (2012). Increased toll-like receptor activity in patients with metabolic syndrome. Diabetes Care.

[29] Poulain-Godefroy O., Le Bacquer O., Plancq P., Lecoeur C., Pattou F., Fruhbeck G., Froguel P. (2010). Inflammatory role of Toll-like receptors in human and murine adipose tissue. Mediators Inflamm..

[30] Nguyen M.T., Favelyukis S., Nguyen A.K., Reichart D., Scott P.A., Jenn A., Liu-Bryan R., Glass C.K., Neels J.G., Olefsky J.M. (2007). A subpopulation of macrophages infiltrates hypertrophic adipose tissue and is activated by free fatty acids via Toll-like receptors 2 and 4 and JNK-dependent pathways. J. Biol. Chem..

[31] Baker R.G., Hayden M.S., Ghosh S. (2011). NF-κB, inflammation, and metabolic disease. Cell Metab..

[32] Hayden M.S., Ghosh S. (2008). Shared principles in NF-kappaB signaling. Cell.

[33] Unger R.H., Orci L. (2000). Lipotoxic diseases of nonadipose tissues in obesity. Int. J. Obes..

[34] Unger R.H., Zhou Y.T. (2001). Lipotoxicity of β-Cells in Obesity and in Other Causes of Fatty Acid Spillover. Diabetes.

[35] Navina S., Acharya C., DeLany J.P., Orlichenko L.S., Baty C.J., Shiva S.S., Durgampudi C., Karlsson J.M., Lee K., Bae K.T. (2011). Lipotoxicity Causes Multisystem Organ Failure and Exacerbates Acute Pancreatitis in Obesity. Sci. Transl. Med..

[36] DeFronzo R.A. (2010). Insulin resistance, lipotoxicity, type 2 diabetes and atherosclerosis: The missing links. The Claude Bernard Lecture 2009. Diabetologia.

[37] Cusi K. (2012). Role of Obesity and Lipotoxicity in the Development of Nonalcoholic Steatohepatitis: Pathophysiology and Clinical Implications. Gastroenterology.

[38] Thomas M.E., Harris K.P.G., Walls J., Furness P.N., Brunskill N.J. (2012). Fatty acids exacerbate tubule interstitial injury in protein-overload proteinuria. Am. J. Physiol. Renal. Physiol..

[39] Rubio M., Gargallo M., Millán A., Moreno B. (2007). Drugs in the treatment of obesity: Sibutramine, orlistat and rimonabant. Public Health Nutr..

[40] De la Garza A.L., Milagro F.I., Bloke N., Campion Martinez J.A. (2011). Natural inhibitors of pancreatic lipase as new players in obesity treatment. Planta Med..

[41] Hardeman W., Griffin S., Johnston M., Kinmonth A.L., Wareham N.J. (2000). Interventions to prevent weight gain: A systematic review of psychological models and behaviour change methods. Int. J. Obes. Relat. Metab. Disord..

[42] Rodgers R.J., Tschöp M.H., Wilding J.P.H. (2012). Anti-obesity drugs: Past, present and future. Dis. Model. Mech..

[43] Cooke D., Bloom S. (2006). The obesity pipeline: Current strategies in the development of anti-obesity drugs. Nat. Rev. Drug Discov..

[44] Sargent B.J., Moore N.A. (2009). New central targets for the treatment of obesity. Br. J. Clin. Pharmacol..

[45] Birari R., Bhutani K. (2007). Pancreatic lipase inhibitors from natural sources: Unexplored potential. Drug Discov. Today.

[46] Sumantran V. (2007). Experimental approaches for studying uptake and action of herbal medicines. Phytother. Res..

[47] Park M.Y., Lee K.S., Sung M.K. (2005). Effects of dietary mulberry, Korean red ginseng, and banaba on glucose homeostasis in relation to PPAR-a, PPAR-c, and LPL mRNA expressions. Life Sci..

[48] Nakayama T., Suzuki S., Kudo H., Sassa S., Nomura M., Sakamoto S. (2007). Effects of three Chinese herbal medicines on plasma and liver lipids in mice fed a high fat diet. J. Ethnopharmacol..

[49] Mayer M.A., Hocht C., Puyo A., Taira C.A. (2009). Recent advances in obesity pharmacotherapy. Curr. Clin. Pharmacol..

[50] Raju J., Gupta D., Rao A.R., Yadava P.K., Baquer N.Z. (2001). TSP foenum-graecum (fenugreek) seed powder improves glucose homeostasis in alloxan diabetic rat tissues by reversing the altered glycolytic, gluconeogenic and lipogenic enzymes. Mol. Cell. Biochem..

[51] Srinivasan K. (2006). Fenugreek (*Trigonella foenum-graecum*): A review of health beneficial physiological effects. Food Rev. Int..

[52] Khalki L., M’hamed S.B., Bennis M., Chait A., Sokar Z. (2010). Evaluation of the developmental toxicity of the aqueous extract from *Trigonella foenum-graecum* (L.) in mice. J. Ethnopharmacol..

[53] Max B. (1999). This and That: The essential pharmacology of herbs and spices. Trends Pharmacol. Sci..

[54] Parry J.W. (1943). The Spice Handbook.

[55] Petit P., Sauvaire Y., Ponsin G., Manteghetti M., Fave A., Ribes G. (1993). Effect of a fenugreek seed extract on feeding behaviour in the rat: Metabolic-endocrine correlates. Pharmacol. Biochem. Behav..

[56] Girardon P., Bessiere J.M., Baccou J.C., Sauvaire Y. (1985). Volatile constituents of fenugreek seeds. Planta Med..

[57] Sharma R.D. (1986). Effect of fenugreek seeds and leaves on blood glucose and serum insulin responses in human subjects. Nutr. Res..

[58] Sauvaire Y., Baccou J.S. (1976). Nutritional value of the proteins of leguminous seed, fenugreek (*Trigonella foenum-graecum* L.). Nutr. Rep. Int..

[59] Baccou J.C., Sauvaire Y., Ollie V., Petit L.J. (1978). L’huile de fenugreec, composition, properties, possibilities d’utilisation dans l’industrie des peintures et vernis. Rev. Fr. Corps Gras.

[60] El-Mahdy A.R., El-Sebaiy L.A. (1985). Proteolytica activity, amino acid composition, protein quality of fermented fenugreek seeds (*Trigonella foenum-graecum*). Food Chem..

[61] Udayasekhara Rao P., Sharma R.D. (1987). An evaluation of protein quality of fenugreek seeds (*Trigonella foenum-graecum*) and their supplementary effects. Food Chem..

[62] Anis M., Aminuddin E. (1985). Estimation of diosgenin in seeds of induced autoploid *Trigonella foenum-graecum*. Fitoterapia.

[63] Brenac P., Sauvaire Y. (1996). Accumulation of sterols and steroidal sapogenins in developing fenugreek pods: Possible biosynthesis in situ. Phytochemistry.

[64] Ghosal S., Srivastava R.S., Chatterjee D.C., Dutta S.K. (1974). Extractives of Trigonella-1. Fenugreekine, a new steroidal sapogenin-peptide ester of *Trigonella foenum-graecum*. Phytochemistry.

[65] Sauvaire Y., Baissac Y., Leconte O., Petit P., Ribes G. (1996). Steroid saponins from fenugreek and some of their biological properties. Adv. Exp. Med. Biol..

[66] Sharma R.D. (1984). Hypocholesterolaemic activity of fenugreek (*T. foenum-graecum*): An experimental study in rats. Nutr. Rep. Int..

[67] Petit P.R., Sauvaire Y.D., Hillaire-Buys D.M., Leconte O.M., Baissac Y.G., Ponsin G.R., Ribes G.R. (1995). Steroid saponins from fenugreek seed: Extraction, purification and pharmacological investigation on feeding behaviour and plasma cholesterol. Steroids.

[68] Mishkinsky J., Joseph B., Sulman F. (1967). Hypoglycaemic effect of trigonelline. Lancet.

[69] Sankara Rao D.S., Deosthale Y.G. (1981). Mineral composition of four Indian food legumes. J. Food Sci..

[70] Hemavathy J., Prabhakar J.V. (1989). Lipid composition of fenugreek (*Trigonella foenum-graecum* L.) seeds. Food Chem..

[71] Varshney I.P., Sharma S.C. (1996). Saponins XXXII *Trigonella foenum-graecum* seeds. J. Indian Chem. Soc..

[72] Gopalan C., Rama Shatsri B.V., Balasubramanyan S.C. (1978). Nutritive Value of Indian Foods.

[73] Sauvaire Y., Girardon P., Baccou J.C., Risterucci A.M. (1984). Changes in growth, proteins and free amino acids of developing seed and pod of fenugreek. Phytochemistry.

[74] Chopra R.N., Chopra I.C., Honda K.L., Kapur L.D. (1982). Chopra’s Indigenous Drugs of India.

[75] Fazli F.R.Y., Hardman R. (1968). The spice, fenugreek (*Trigonella foenum-graecum*): Its commercial varieties of seed as a source of diosgenin. Trop. Sci..

[76] Amin R., Abdul-Ghani A.S., Suleiman M.S. (1987). Intestinal absorption. In: Proceedings of the 47th Annual Meeting of the American Diabetes Association (Indianapolis U.S.A.). Diabetes.

[77] Al-Meshal I.A., Parmar N.S., Tariq M., Aqeel A.M. (1985). Gastric anti-ulcer activity in rats of *Trigonella foenum-graecum* (Hu-Lu-Pa). Fitoterapia.

[78] Singhal P.C., Gupta R.K., Joshi L.D. (1982). Hypocholesterolaemic effect of *Trigonella foenum-graecum* (Methi). Curr. Sci..

[79] Sharma R.D., Sarkar A., Hazar D.K., Misra B., Singh J.B., Maheshwari B.B. (1996). Toxicological evaluation of fenugreek seeds: A long term feeding experiment in diabetic patients. Phytother. Res..

[80] Grover J.K., Yadav S., Vats V. (2002). Medicinal plants of India with anti-diabetic potential. J. Ethnopharmacol..

[81] Srinivasan S., Stevens M., Wiley J.W. (2000). Diabetic peripheral neuropathy: Evidence for apoptosis and associated mitochondrial dysfunction. Diabetes.

[82] Broca C., Gross R., Petit P., Sauvaire Y., Manteghetti M., Tournier M., Masiello P., Gomis R., Ribes G. (1999). 4-Hydroxyisoleucine: Experimental evidence of its insulinotropic and antidiabetic properties. Am. J. Physiol..

[83] Broca C., Manteghetti M., Gross R., Baissac Y., Jacob M., Petit P., Sauvaire Y., Ribes G. (2000). 4-Hydroxyisoleucine: Effects of synthetic and natural analogues on insulin secretion. Eur. J. Pharmacol..

[84] Yadav U.C.S., Moorthy K., Baquer N.Z. (2004). Effects of sodium orthovanadate and *Trigonella foenum-graecum* seeds on hepatic and renal lipogenic enzymes and lipid profile during alloxan diabetes. J. Biosci..

[85] Yadav U.C.S., Moorthy K., Baquer N.Z. (2005). Combined Treatment of Sodium orthovanadate and Momordica charantia fruit extract prevents alterations in lipid profile and lipogenic enzymes in alloxan diabetic rats. Mol. Cell. Biochem..

[86] Flammang A., Cifone M., Erexson G., Stankowski L. (2004). Genotoxicity testing of a fenugreek extract. Food Chem. Toxicol..

[87] Fuller S., Stephens J.M. (2015). Diosgenin, 4-Hydroxyisoleucine, and Fiber from Fenugreek: Mechanism of Actions and potential Effects on Metabolic Syndrome. Adv. Nutr..

[88] Gong J., Fang K., Dong H., Wang D., Hu M., Lu F. (2016). Effect of fenugreek on hyperglycaemia and hyperlipidemia in diabetes and prediabetes: A meta-analysis. J. Ethnopharmacol..

[89] Hajimehdipoor H., Sadat-Ebrahimi S.E., Izaddoost M., Amin G.R., Givi E. (2008). Identification and quantitative determination of blood lowering sugar amino acid in Fenugreek. Planta Med..

[90] Narender T., Puri A., Khaliq T., Saxena R., Bhatia G., Chandra R. (2006). 4-Hydroxyisoleucine an unusual amino acid as antidyslipidemic and antihyperglycemic agent. Bioorg. Med. Chem. Lett..

[91] Mehrafarin A., Qaderi A., Rezazadeh S.H., Naghdi-Badi H., Noormohammadi G.H., Zand E. (2010). Bioengineering of important secondary metabolites and metabolic pathways in fenugreek (*Trigonella foenum-graecum* L.). J. Med. Plant..

[92] Sauvaire Y., Petit P., Broca C., Manteghetti M., Baissac Y., Fernandez-Alvarez J., Gross R., Roye M., Leconte A., Gomis R. (1998). 4-Hydroxyisoleucine: A novel amino acid potentiator of insulin secretion. Diabetes.

[93] Fowden L., Pratt H.M.S., Mith A. (1973). 4-Hydroxyisoleucine from seed of *Trigonella foenum-graecum*. Phytochemistry.

[94] Skaltsa H., Petropoulos G.A. (2002). Chemical constituents. Fenugreek—The genus Trigonella.

[95] Broca C., Breil V., Cruciani-Guglielmacci C., Manteghetti M., Rouault C., Derouet M., Rizkalla S., Pau B., Petit P., Ribes G. (2004). Insulinotropic agent ID-1101 (4-hydroxyisoleucine) activates insulin signaling in rat. Am. J. Physiol. Endocrinol. Metab..

[96] Acharya S.N., Thomas J.E., Basu S.K. (2008). Fenugreek, an alternative crop for semi-arid regions of North America. Crop Sci..

[97] Baquer N.Z., Taha A., Kumar P., McLean P., Cowsik S.M., Kale R.K., Singh R., Sharma D. (2009). A metabolic and functional overview of brain aging linked to neurological disorders. Biogerontology.

[98] Mohammad S., Taha A., Akhtar K., Bamezai R.N., Baquer N.Z. (2006). In vivo effect of *Trigonella foenum-graecum* on the expression of Pyruvate kinase, Phosphoenolpyruvate carboxykinase and distribution of glucose transporter (GLUT4) in alloxan diabetic rats. Can. J. Physiol. Pharm..

[99] Shukla P., Rangari V. (2015). Enhancement of anti-diabetic activity of 4-hydroxyisoleucine in combination with natural bioavailability enhancers. Int. J. Pharm. Pharm. Sci..

[100] Haeri M.R., Izaddoost M., Ardekani M.R.S., Nobar M.R., White K.N. (2009). The effect of fenugreek 4-hydroxyisoleucine on liver function biomarkers and glucose in diabetic and fructose-fed rats. Phytother. Res..

[101] Singh A.B., Tamarkar A.K., Narender T., Srivastava A.K. (2010). Antihyperglycaemic effect of an unusual amino acid (4-hydroxyisoleucine) in C57BL/KsJ-db/db mice. Nat. Prod. Res..

[102] Haeri M.R., Limaki H.K., White C.J., White K.N. (2012). Non-insulin dependent anti-diabetic activity of (2*S*,3*R*,4*S*)4-hydroxyisoleucine of fenugreek (*Trigonella foenum-graecum*) in streptozotocin-induced type I diabetic rats. Phytomedicine.

[103] Jaiswal N., Maurya C.K., Venkateswarlu K., Sukanya P., Srivastava A.K., Narender T., Tamrakar A.K. (2012). 4-Hydroxyisoleucine stimulates glucose uptake by increasing surface GLUT4 level in skeletal muscle cells via phosphatidylinositol-3-kinase-dependent pathway. Eur. J. Nutr..

[104] Maurya C.K., Singh R., Jaiswal N., Venkateswarlu K., Narender T., Tamrakar A.K. (2014). 4-Hydroxyisoleucine ameliorates fatty acid-induced insulin resistance and inflammatory response in skeletal muscle cells. Mol. Cell. Endocrinol..

[105] Yu H., Wu M., Lu F.R., Xie J., Zheng N., Qin Y., Gao F., Du W., Jian L.M. (2013). Effect of trigonella foenum-graecum 4-hydroxyisoleucine on high-glucose induced insulin resistance in 3T3-L1 adipocytes of mice. Chin. J. Integr. Tradit. West. Med..

[106] Zafar M.I., Du W., Cai Q., Shafqat R.A., Lu F. (2015). 4-Hydroxyisoleucine improves insulin resistance in HepG2 cells by decreasing TNF-α and regulating the expression of insulin signal transduction proteins. Mol. Med. Rep..

[107] Olaiya O.C., Soetan O.K. (2014). A review of the health benefits of fenugreek (*Trigonella foenum-graecum* L.): Nutritional, Biochemical and pharmaceutical perspectives. Am. J. Soc. Issues Humanit..

